# Residential location of people with chronic spinal cord injury: the importance of local health care infrastructure

**DOI:** 10.1186/s12913-018-3449-3

**Published:** 2018-08-22

**Authors:** Elias Ronca, Thekla Brunkert, Hans Georg Koch, Xavier Jordan, Armin Gemperli

**Affiliations:** 1 Swiss Paraplegic Research, Nottwil, Switzerland; 2 Department of Health Sciences and Health Policy, University of Lucerne, Lucerne, Switzerland; 3 Nursing Science (INS), Department Public Health (DPH), Faculty of Medicine, University of Basel, Basel, Switzerland; 4 Applied Knowledge Transfer, Swiss Paraplegics Association, Nottwil, Switzerland; 5 Spinal Cord Unit, Clinique Romande de Réadaptation SUVACare, Sion, Switzerland

**Keywords:** Residential location, Access, Health care infrastructure, Spinal cord injury, Disability, Environmental barriers

## Abstract

**Background:**

People with spinal cord injury (SCI) suffer from complex secondary health conditions and rely on specialized health care services, which are often centralized and difficult to reach for individuals living in remote areas. As a consequence, they might move to regions where they expect better access to care. The aims of this study were: 1) to identify regions where people with SCI live compared with the general population, 2) to examine whether their choice of residence is related to the availability of local health care infrastructure, and 3) to ascertain determinants of their consideration to change residence when aging.

**Methods:**

This study used information from a nationwide Swiss SCI cohort and inpatient hospital discharge data. To detect clusters in the distribution of people with chronic SCI in Switzerland, a spatial cluster detection test was conducted using the normative population of a region as offset. To identify associations between the residential location of people with SCI and infrastructure variables, a negative binomial model was set up at a regional level with the frequency of people with SCI as outcome, geographical indicators as explanatory variables, and the normative population as offset. Determinants of the consideration to change residence when aging were investigated using logistic regression models.

**Results:**

People with SCI were not living equally distributed among the normative population, but clustered in specific areas. They were more likely than the general population to reside close to specialized SCI centers, in areas with a high density of outpatient physicians, and in urban regions. People with SCI living in rural areas were more likely to consider relocating when aging than those living in urban areas. However, only a few people with SCI considered moving closer to specialized centers when such a move required crossing language barriers.

**Conclusions:**

Good access to appropriate health care services and amenities of daily life seems to play such an important role in the lives of people with SCI that they are willing to choose their residential location based on local availability of appropriate health care services.

## Background

A spinal cord injury (SCI) causes damage to the somatic and autonomic nervous systems and typically leads to lifelong complete or incomplete paralysis of the locomotor system and dysfunction of organs innervated from below the lesion level [1]. This results in mobility limitations and a wide range of frequent secondary health complications in affected people [2]. Because SCI is a rare event and its secondary health complications are generally complex, many health care professionals lack the necessary expertise to adequately treat people with SCI [3, 4]. Thus, many people with SCI bypass local health care providers to receive treatment at specialized centers [5]. The farther people with SCI live from those centers, the more unmet health care needs they report, especially when they also have insufficient access to long-distance transportation [6]. Similarly, access to appropriate primary and specialized health care services as well as community reintegration is reportedly more difficult for people with SCI living in rural areas than for those living in cities [7–9].

Maintaining the long-term health status of people with SCI requires access to both general and specialist services [10]. Different strategies have been employed to enhance access to specialized health care services in underserved regions, such as the provision of outreach services, telemedicine, or the introduction of small outpatient clinics [8, 9]. Given that the proximity to available community resources directly affects the well-being of people with neurological disability by influencing access to needed resources [11], it may be possible that people with SCI relocate to where they expect fewer environmental barriers and better access to adequate health care services. In fact, a Canadian study by Glennie et al. found that 1 year after a traumatic SCI, 13 % of people with SCI have moved from a rural to an urban setting [12]. People with SCI in their forties reportedly have a life space (a measure of where a person goes, the frequency of going there, and the dependency in getting there) comparable to the average reported for community-dwelling elderly people with a mean age of 75 years [13]. Close proximity to required health care infrastructure might be even more important for people with high-level SCI (tetraplegia) as their life space was shown to be smaller than that of people with lower-level lesions (paraplegia) [13].

So far, research investigating appropriate housing for people with neurological impairment has focused on features related to housing design and neighborhood setting, with limited or no focus on housing location [11]. Understanding whether people with SCI live equally distributed among the able-bodied population or choose their residential location based on specific preferences is crucial to the development of suitable housing solutions and health care resource allocation. Therefore, the specific aims of this study were: 1) to identify regions where people with SCI were more likely to live compared to the general population, and to examine whether particularly vulnerable groups, such as the elderly or tetraplegics were more likely to settle in these regions, 2) to study whether the choice of residence was related to the availability of local health care infrastructure, and 3) to ascertain determinants of the consideration to change residence when aging.

## Methods

### Study population

This study relied on location and socio-demographic information of a nationwide SCI cohort study (SwiSCI) [14] and hospital discharge data. Participants in SwiSCI were predominantly recruited through three out of four specialized SCI-rehabilitation centers and institutions that are connected to these centers, such as the Swiss Paraplegic Association (SPA) and ParaHelp. Due to a known underrepresentation of elderly people and those without SPA membership in SwiSCI [15, 16], another study population consisting of patients with an SCI diagnosis identified from hospital discharge data [17] was included in the current study.

The SwiSCI study aims to provide reliable epidemiologic data on people with SCI living in Switzerland and includes participants with chronic SCI, after discharge from first rehabilitation, who are 16 years or older and permanently living in Switzerland. People with congenital conditions leading to SCI (e.g., spina bifida), new SCI in the context of palliative care, neurodegenerative disorders (e.g., multiple sclerosis, amyotrophic lateral sclerosis), and Guillain-Barré syndrome are excluded [14]. SwiSCI consists of three questionnaires administered between September 2011 and March 2013. The first two questionnaires query socio-demographics and lesion characteristics. The third questionnaire includes information on health services and aging.

This study was approved by the ethics committee of northwest/central Switzerland, and by the care and patient organizations where the contact addresses were collected. All the necessary administrative permissions to access and use the data in the current study were obtained. The study used basic information (lesion level, age, place of residence) of 3054 registered individuals. The place of residence was referenced to medical statistics (Medstat) regions by the SwiSCI study center. Medstat regions are geographical units constructed from zip code regions large enough to guarantee anonymization [18]. Information on whether people with SCI are more likely to relocate when older was derived from the third questionnaire of the SwiSCI community survey on health services and aging [14, 19]. Socio-demographics and lesion characteristics were derived from the first two questionnaires.

The hospital discharge data comprised nearly all (99%) inpatient hospitalizations that occurred in Switzerland [17]. Hospitalization records were irreversibly anonymized by the data providers, with unique IDs assigned to the patients. The present study used data from the years 2007–2011 and the following variables: Date of hospitalization, age, place of residence (Medstat), and International Statistical Classification of Diseases and Related Health Problems, 10th Revision, German Modification (ICD-10-GM) codes. The ICD-10-GM codes were used 1) to identify people with SCI (paraplegia and tetraplegia: G82), 2) to identify those with a high-level lesion (tetraplegia: G82.3-G82.5), and 3) to exclude people with health conditions that lead to exclusion from the SwiSCI study. Only the latest available record of every patient was kept for further analyses.

By comparing the number of hospitalizations and the normative population of each Medstat region, and by comparing the number of hospitalizations over the studied years, irregularities in coding of the place of residence were detected in 14 of 705 Medstat regions. This incorrect coding of place of residence in the hospital discharge data has been described before [20]. Issues in the data were handled by combining five and seven neighboring Medstat regions, respectively, into two new regions and by reassigning hospitalizations from another region to one of the two created regions.

### Characteristics of regions

Information about the degree of urbanization and language were provided by the Swiss Federal Statistical office on a municipality level [21] and were mapped to Medstat regions. The degree of urbanization was defined as equivalent to the enclosed municipality with the highest degree of urbanization. If Medstat regions contained municipalities with different local languages, then the language spoken by the majority of the population was adopted. Information about the number of physicians working in the outpatient and inpatient sector was provided by the Swiss Medical Association [22] on a zip code level and was mapped to Medstat regions. Information about the density of home care professionals was provided on a cantonal level [23] and mapped to Medstat regions. Car travel times from the centroid of the residential Medstat region to the closest of four specialized SCI centers were estimated using the Google Maps Directions API [24]. Normative population information was acquired on a hectare level [25] and mapped to Medstat regions.

### Statistical analysis

To detect and evaluate the statistical significance of clusters in the distribution of people with SCI in Switzerland, a spatial cluster detection test was conducted using SaTScan software version 9.4.4 [26]. The number of people with SCI living in a Medstat region was modeled as a discrete Poisson distribution to allow for different densities of background population [27]. The normative population of a Medstat region was used as offset. SaTScan was set to find spatial clusters of a maximum size of 25% of the SCI population. The significance of found clusters was assessed at a *p*-value < 0.01 and performed by 999 Monte Carlo realizations. To identify clusters in the distribution of people with SCI who were older than 65, only the part of the normative population that was older than 65 was used as offset.

To identify associations between the residential location of people with SCI and infrastructure variables, a negative binomial model was set up at the Medstat level with the frequency of people with SCI as outcome, geographical indicators as explanatory variables, and the normative population as offset. To analyze associations in people with SCI who were older than 65, again only the part of the normative population that was older than 65 was used as offset. A negative binomial model was chosen based on a preliminary Chi-square test that revealed overdispersion in the data.

Determinants of the consideration to relocate residence when aging (“considering residential relocation when aging (or not)”) and relocate closer to a specialized SCI center (“considering moving close to a specialized SCI center when aging (or not)”) were investigated using two logistic regression models. The explanatory variables used in both models were age, sex, severity of injury (complete/incomplete, high−/low-level lesion, cause of injury (traumatic/non-traumatic), language region (German, French, Italian), and the degree of urbanization of the place of residence (urban/suburban/rural).

Statistical analyses and data management were performed using the statistical software R version 3.4.1 (R Foundation for Statistical Computing, Vienna, Austria).

## Results

A total of 3054 individuals with SCI were identified from SwiSCI, and an additional 10,456 were identified from hospital discharge data (Table 1). The median number of people with SCI, per approximately ten thousand inhabitants of a Medstat region, was four from the SwiSCI data and 12 from hospital discharge data. In three out of four Medstat regions, individuals with SCI could reach a specialized SCI center within a driving time of 70 min by car.

**Table 1:** Characteristics of residential (Medstat) regions of people with SCI

Properties of Medstat regions	SwiSCI data 705 Medstat regions	Hospital discharge data 695^a^ Medstat regions
Number of inhabitants — median (Q1, Q3)		
General population	10,113 (7906 — 13,505)	10,151 (7910 — 13,521)
People with SCI		
All	4 (2 — 6)	12 (8 — 18)
Tetraplegic	1 (0 — 2)	4 (3 — 7)
Older than 65 years	1 (0 — 2)	5 (3 — 9)
Degree of urbanization, n (%)		
Urban	144 (20.4)	140 (20.1)
Suburban	340 (48.2)	337 (48.5)
Rural	221 (31.3)	218 (31.4)
Language region, n (%)		
German	502 (71.2)	492 (70.8)
French	166 (23.5)	166 (23.9)
Italian	37 (5.2)	37 (5.3)
Number of physicians— median (Q1, Q3)		
Outpatient sector	10 (5 — 23)	11 (5 — 25)
Inpatient sector	6 (2 — 17)	6 (2 — 17)
Number of home care professionals per 10,000 inhabitants — median (Q1, Q3)	19 (13 — 29)	19 (13 — 29)
Driving time to the closest specialized SCI center in minutes – median (Q1, Q3)	54 (33 — 76)	54 (34 — 76)

A total of 3054 and 10,456 individuals with SCI were identified from SwiSCI and hospital discharge data, respectively

There were no missing values

*Abbreviations*: *Medstat regions* Medical statistics regions, *Q1* lower quartile, *Q3* upper quartile, *SCI* spinal cord injury, *SwiSCI* Swiss Spinal Cord Injury Cohort Study

^a^Certain regions had to be merged to overcome issues with imprecise assignments of patient addresses to Medstat regions

**Fig. 1:**
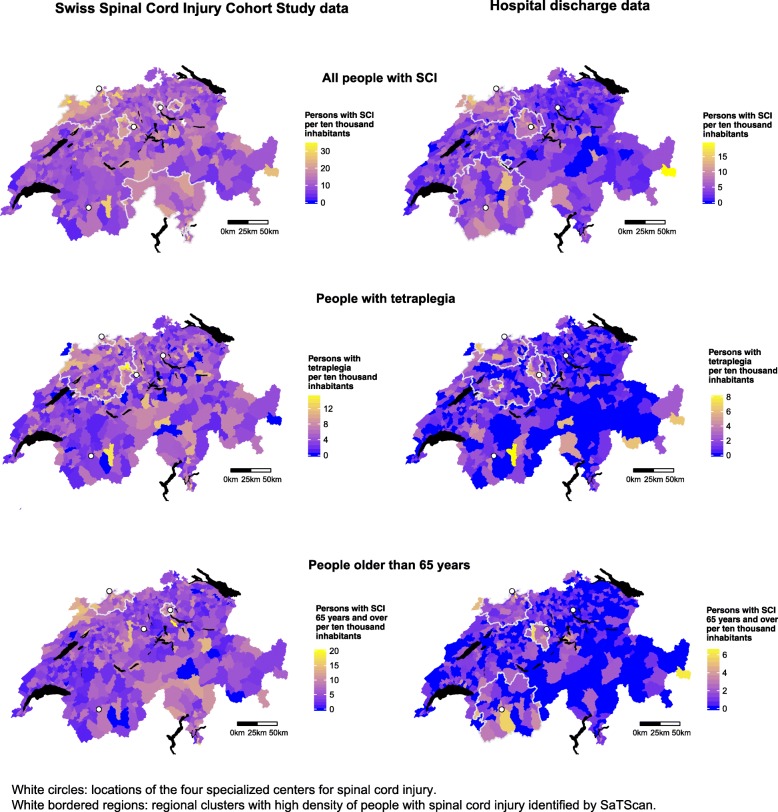
Preferred residential areas of people with spinal cord injury*. Note.* This figure was created by the first author (ER) using the ggplot2 R package [39]

People with SCI were not living equally distributed among the total population, but clustered around specialized SCI centers and within larger cities (Fig. 1). All but one cluster identified from the SwiSCI data were located around the three specialized rehabilitation centers that were involved in recruiting participants for the SwiSCI study. People with SCI identified from hospital discharge data were also living in clusters around the easternmost SCI center, which was not involved in recruiting SwiSCI participants. The hospital discharge data also revealed a cluster in the Italian-speaking region that lies in the southeastern part of Switzerland. The clusters indicating the residential location of people with tetraplegia were almost identically shaped for both samples. These clusters were situated between two large specialized SCI centers, enclosed two of the four largest Swiss cities, and covered German- and French-speaking regions. Elderly people with SCI were mostly living in clusters around SCI centers close to urban areas.

If measured in terms of the normative population of a (Medstat) region, people with SCI were more likely to live in urban than in suburban or rural regions (Table 2). This trend was found in both the SwiSCI and the hospital population but was more pronounced in the latter case. Similarly, a high propensity to live in regions with a greater density of outpatient physicians was found in both datasets. This effect was slightly stronger in elderly people with SCI than in the general SCI population. No clear associations within and across both samples were found between the densities of inpatient physicians or home care professionals in a region and the number of individuals with SCI living in that region in relation to the total population. A strong correlation was found between the driving time to the closest specialized SCI center and the likelihood to live in a specific region. Individuals identified through SwiSCI were more likely to be living in regions close to specialized SCI centers than in regions further from these centers.

**Table 2:** Relative number of inhabitants with SCI, by characteristics of region

	SwiSCI data			Hospital discharge data		
	All SCI cases	Tetraplegics	> 65 years of age	All SCI cases	Tetraplegics	> 65 years of age
	IRR (95% CI)	IRR (95% CI)	IRR (95% CI)	IRR (95% CI)	IRR (95% CI)	IRR (95% CI)
Degree of urbanization, ref.: Rural						
Suburban	1.06 (1.02 — 1.10)^*^	1.14 (1.07 — 1.21)^*^	1.17 (1.08 — 1.26)^*^	1.16 (1.13 — 1.20)^*^	1.11 (1.06 — 1.15)^*^	1.17 (1.12 — 1.22)^*^
Urban	1.39 (1.31 — 1.47)^*^	1.47 (1.36 — 1.59)^*^	1.22 (1.11 — 1.35)^*^	1.88 (1.80 — 1.96)^*^	1.84 (1.75 — 1.95)^*^	1.82 (1.73 — 1.93)^*^
Language, ref.: German						
French	1.10 (1.04 — 1.16)^*^	0.90 (0.84 — 0.97)^*^	1.19 (1.09 — 1.30)^*^	0.89 (0.86 — 0.93)^*^	0.81 (0.77 — 0.86)^*^	0.86 (0.82 — 0.91)^*^
Italian	0.96 (0.86 — 1.06)	0.75 (0.64 — 0.88)^*^	0.74 (0.60 — 0.91)^*^	1.13 (1.05 — 1.22)^*^	1.06 (0.97 — 1.17)	1.25 (1.14 — 1.38)^*^
Number of physicians in outpatient sector per 10,000 inhabitants, ref.: < 5						
5- < 10	1.18 (1.11 — 1.25)^*^	1.14 (1.05 — 1.25)^*^	1.45 (1.29 — 1.62)^*^	1.21 (1.16 — 1.26)^*^	1.22 (1.16 — 1.30)^*^	1.32 (1.24 — 1.40)^*^
10 - < 20	1.34 (1.26 — 1.42)^*^	1.42 (1.30 — 1.55)^*^	1.45 (1.29 — 1.63)^*^	1.33 (1.27 — 1.39)^*^	1.26 (1.19 — 1.33)^*^	1.53 (1.44 — 1.62)^*^
20+	1.55 (1.44 — 1.67)^*^	1.49 (1.34 — 1.66)^*^	2.03 (1.78 — 2.33)^*^	1.69 (1.60 — 1.78)^*^	1.61 (1.50 — 1.72)^*^	1.91 (1.78 — 2.05)^*^
Number of physicians in inpatient sector per 10,000 inhabitants, ref.: < 3						
3- < 6	1.01 (0.96 — 1.06)	0.94 (0.87 — 1.01)	0.95 (0.87 — 1.05)	1.12 (1.08 — 1.16)^*^	1.03 (0.98 — 1.08)	1.16 (1.10 — 1.22)^*^
6- < 15	0.96 (0.91 — 1.01)	0.94 (0.87 — 1.01)	0.99 (0.90 — 1.09)	1.12 (1.07 — 1.16)^*^	1.12 (1.07 — 1.17)^*^	1.20 (1.14 — 1.26)^*^
15+	0.92 (0.86 — 0.98)^*^	0.94 (0.87 — 1.03)	0.99 (0.89 — 1.11)	1.09 (1.04 — 1.14)^*^	1.09 (1.03 — 1.15)^*^	1.19 (1.12 — 1.26)^*^
Average number of home care professionals per 10,000 inhabitants, ref.: < 15						
15 - < 20	0.79 (0.75 — 0.84)^*^	0.76 (0.71 — 0.82)^*^	0.81 (0.73 — 0.89)^*^	1.07 (1.03 — 1.11)^*^	1.00 (0.95 — 1.05)	1.18 (1.12 — 1.25)^*^
20+	1.04 (0.99 — 1.10)	1.15 (1.06 — 1.24)^*^	1.31 (1.18 — 1.44)^*^	1.10 (1.05 — 1.14)^*^	1.10 (1.04 — 1.16)^*^	1.24 (1.17 — 1.31)^*^
Driving time to the closest specialized SCI center, ref. > 90 min						
< 30 min	1.82 (1.70 — 1.96)^*^	1.76 (1.59 — 1.95)^*^	2.69 (2.37 — 3.05)^*^	1.44 (1.37 — 1.52)^*^	1.55 (1.45 — 1.66)^*^	1.50 (1.40 — 1.61)^*^
30–60 min	1.23 (1.15 — 1.32)^*^	1.13 (1.02 — 1.25)	1.56 (1.38 — 1.76)^*^	1.13 (1.07 — 1.19)^*^	1.15 (1.08 — 1.23)^*^	1.12 (1.05 — 1.20)^*^
> 60–90 min	1.13 (1.06 — 1.21)^*^	1.12 (1.02 — 1.23)	1.26 (1.12 — 1.42)^*^	0.97 (0.92 — 1.02)	1.04 (0.98 — 1.11)	0.97 (0.91 — 1.03)

Analysis based on a negative binomial model with the frequency of people with SCI per region as outcome, regional indicators as explanatory variables, and the age-specific normative population per region as offset

*Abbreviations*: *SCI* spinal cord injury, *IRR* incidence rate ratio, *CI* confidence interval, *SwiSCI* Swiss Spinal Cord Injury Cohort Study

^*^Incidence rate ratio with *p* < 0.05

Three out of five SwiSCI participants (60%) who completed the third SwiSCI questionnaire about aging (*N* = 492) reported considering moving out of their current municipality to a more age-appropriate residential location and slightly more than one in four people with SCI (28%) reported considering moving closer to an SCI center when getting older. The consideration given to moving out of the residential municipality and the consideration given to moving close to an SCI center decreased with increasing age (Table 3). Although not statistically significant at a five-percent significance level, there was a trend towards a higher propensity to settle close to specialized SCI centers among people with more severe lesions.

**Table 3:** Determinants of the consideration to relocate residence when aging

	Residential relocation when aging	Moving close to a specialized SCI center when aging
	OR (95% CI)	OR (95% CI)
Age, ref.: 16–45 years		
46–60 years	0.53 (0.29 — 0.96)^*^	1.23 (0.73 — 2.06)
61–75 years	0.27 (0.15 — 0.47)^*^	0.72 (0.41 — 1.23)
76+ years	0.18 (0.07 — 0.42)^*^	0.22 (0.05 — 0.71)^*^
Sex, Female	1.04 (0.66 — 1.67)	0.87 (0.54 — 1.40)
Severity, ref.: Incomplete paraplegia		
Incomplete tetraplegia	0.99 (0.56 — 1.75)	1.38 (0.78 — 2.43)
Complete paraplegia	0.96 (0.57 — 1.62)	1.37 (0.82 — 2.31)
Complete tetraplegia	0.74 (0.35 — 1.62)	1.89 (0.88 — 3.99)
Cause of SCI, ref.: Traumatic	0.72 (0.43 — 1.22)	1.10 (0.62 — 1.92)
Language region, ref.: German		
French	1.48 (0.91 — 2.46)	0.61 (0.36 — 0.99)^*^
Italian	0.40 (0.16 — 1.00)	0.21 (0.03 — 0.74)^*^
Residential area, ref.: Urban		
Suburban	2.00 (1.23 — 3.28)^*^	1.15 (0.69 — 1.94)
Rural	2.19 (1.25 — 3.89)^*^	0.96 (0.54 — 1.71)

Analysis based on logistic regression adjusted for all determinants listed

*Abbreviations*: *SCI* spinal cord injury, *OR* odds ratio, *CI* confidence interval

^*^Odds ratio with *p* < 0.05

People with SCI from non-German-speaking regions were less likely to consider moving close to SCI centers. For example, individuals from the Italian-speaking parts of Switzerland were five times less likely (odds ratio (OR) 0.21, 95%-confidence interval (CI) 0.03–0.74) to consider moving close to an existing SCI center than people from German-speaking parts of Switzerland. Individuals with SCI living in urban areas were about half as likely to consider moving to another residential location than individuals from suburban (OR 2.00, CI 1.23–3.28) or rural (OR 2.19, CI 1.25–3.89) areas.

## Discussion

People with SCI do not live equally distributed among the normative population, but clustered in and around larger cities and close to specialized SCI centers. Good access to appropriate health care seems to play such an important role in the lives of people with SCI that they are willing to choose their residential location based on the availability of appropriate health care. This is interesting, particularly since the health care supply in Switzerland is generally high and a doctor is never really far away regardless of where a person lives (4.0 physicians per 1000 residents; 99.8% of the population reaches a general hospital by car within 30 min, 94.0% within 15 min; 75% reach a specialized SCI center within 75 min) [28].

People with disabilities generally perceive rural areas to be less sensitive to disability access issues than urban areas [29], particularly those who rely on the services of large medical centers [30]. Such experiences might explain why people with SCI in Switzerland are more likely to live in urban areas than able-bodied people. In addition to a higher supply of health care services, there might be other reasons why people with SCI prefer to live in urban areas. Wright et al. found in their literature review about optimal housing features for adults with neurological disabilities that housing is most convenient when located within walking (or wheeling) distance of local amenities, and local services such as cafés, shopping centers, churches, and banks, as well as physically accessible, affordable, and reliable public transport [11]. All those amenities are most likely to be found in close proximity in urban areas.

Specialized services are the favored source of care of most people with SCI [31, 32], and people with SCI are willing to travel more often and longer distances to obtain specialized care than the general population [33]. Only when car travel time to a specialized center was 70 min or longer did a majority of people with SCI consider obtaining less specialized services [6]. The closer a region is located to a specialized center the more likely it is for people with SCI to live in that region. Reasons to live close to specialized centers might also be the availability of a broad spectrum of sports facilities tailored to the needs of people with SCI, wheelchair workshops, the supply of medical devices, and incontinence supplies. However, even though proximity to specialized care seems important to people with SCI, they are not willing to leave their language region to live closer to the necessary services. Therefore, it is suggested that care be provided in the patients’ mother tongue whenever possible, and that specialized services be made available in every language region of a country. Otherwise, language barriers appear to hinder access to specialized services.

Besides living close to highly specialized SCI centers, it also seems important to people with SCI to have a wide array of outpatient services nearby. People with SCI rely on various outpatient services [28] which they use much more frequently than the general population [34, 35]. The current study has demonstrated a positive correlation between the density of outpatient physicians practicing in a region and the propensity of people with SCI to live there. No similar correlation between the propensity to live in a region and the density of home care professionals or physicians working in the inpatient sector was found. The density of home care professionals was available only on a cantonal level. This information is imprecise and might not allow for sound ecological regression. A study by LaVela et al. has shown that people with SCI bypass local hospitals to obtain care from specialized centers [5]. People with SCI who need inpatient hospital care might prefer to take the extra effort to travel to the more distant SCI centers instead of obtaining inpatient hospital care from general hospitals nearby. This might explain why people with SCI were not more likely to be living in regions with a high density of inpatient physicians than the general population.

This study confirmed a potential underrepresentation of elderly people in the SwiSCI study by comparing the SwiSCI sample to the sample of people with SCI identified from hospital discharge data. However, this might be explained partly by the fact that elderly people are at higher risk for hospitalization, and are therefore more likely to appear in the hospital discharge data. The same might be true for individuals with tetraplegia, who were more prevalent in the hospital data. Furthermore, high lesion levels might be better recognizable as SCI and therefore more likely to be coded as such.

The consequences of the non-involvement of the easternmost SCI center in recruiting participants for the SwiSCI study were clearly seen in the data. This resulted in an underrepresentation of individuals with SCI from the area, illustrated by the absence of clusters around and near this SCI center in the SwiSCI data. Another cluster that is present in the hospital discharge data but not in the SwiSCI data is in the Italian-speaking part of Switzerland. As there is no SCI center present in the Italian-speaking region, people from this region are likely also underrepresented in the SwiSCI data.

Notwithstanding the benefits of moving to locations with better access to services, there are good reasons for staying in one’s current residential location. Social support is essential in achieving community reintegration and maintaining autonomy and independence [11]. Such conditions are normally found in a familiar environment near friends, relatives, including children, and the workplace. All these factors must be considered when planning new outreach services, specialized centers, and housing for people with SCI or disabilities in general.

### Limitations

This study was subject to several limitations. First, due to the cross-sectional study design, it was not clear whether people changed their residential location after the onset of SCI or whether SCI incidences were higher for certain regions. More than one in five (21%) people with SCI in Switzerland have non-traumatic causes for SCI (e.g. cancer, infection, inflammation) [19], which are unlikely to be related to the residential location. The most frequent causes of traumatic SCI in Switzerland are car or motorcycle crashes, skiing or paragliding injuries, as well as tripping or falling from trees or ladders [36]. It is unlikely that these and other reported causes for traumatic SCI are more likely in urban regions, in regions with a high density of outpatient physicians, or close to specialized SCI centers. Second, it was not possible to infer the onset of SCI from hospital discharge data since data availability was limited to records from the last 5 years. Thus, it cannot be conclusively clarified whether enough time has passed between the onset of SCI and data collection for relocation to be studied. However, the SwiSCI population has a median time since injury of 14 years and three out of four participants had been living with an SCI for six or more years before answering the survey. Furthermore, Glennie et al. showed that 13 % of their study participants have relocated as early as 1 year after SCI [12]. Therefore, it seems reasonable to assume that there was enough time for people with SCI to move between the onset of SCI and data collection. Third, some individuals with SCI are working for SCI centers or related institutions close to SCI centers. For these people, the job, rather than good access to care, might have been the reason for moving close to specialized SCI centers. Yet, it is unlikely that these individuals are responsible for the much higher probability of people with SCI to live within a 30-min drive time from a specialized SCI center than farther away. Fourth, hospital discharge data is not collected for research purposes, and studies have demonstrated inaccurate coding of SCI [37, 38]. Fifth, hospital discharge data only includes people who were hospitalized in an inpatient setting. People who were treated in an outpatient setting or received no treatment at all were not covered by the hospital discharge data. Another limitation related to the hospital data concerns the erroneous referencing of residential places in 12 of 706 Medstat regions. Even though we tried to account for the errors, it is not clear to what degree the corrected residential locations still deviated from the real ones. Due to the relatively low number of affected areas (12 of 706), the erroneous referencing of location is not expected to a have significant effect on the results.

## Conclusion

People with SCI were more likely to live in urban regions, in areas with a high density of outpatient physicians, and close to specialized SCI centers than the general population. Good access to appropriate health care seems to play such an important role in the lives of people with SCI that they are willing to choose their residential location based on local health care availability. This is different in people with SCI who must pass a language barrier to live closer to needed services. These people were much less likely to be willing to settle close to specialized SCI centers. Therefore, it is suggested that care be provided in the patients’ mother tongue whenever possible, and that specialized services be made available in every language region of a country. Further spatial studies relating to people with SCI will need to consider that individuals with SCI are not living equally distributed among the normative population but preferentially live in places with appropriate health care and public infrastructure.

## Abbreviations

CI: Confidence Interval
ICD-10-GM: International Statistical Classification of Diseases and Related Health Problems, 10th Revision, German Modification
IRR: Incidence Rate Ratio
Medstat: Medical statistics
OR: Odds Ratio
Q1, Q3: Lower, Upper Quartile
SCI: Spinal Cord Injury
SPA: Swiss Paraplegic Association
SwiSCI: Swiss Spinal Cord Injury Cohort Study
